# Evaluation of *Trichoderma* bio-control agents and pre-cultivation seed treatments for the control of *Cephalosporium maydis* causing late wilt in maize (*Zea mays* L.)

**DOI:** 10.1186/s12870-025-06881-4

**Published:** 2025-07-02

**Authors:** Abeer A. Ahmed, Hend T. Eid, Heba M. Fatouh, Rania A. Saleh, Hend Mohammad Saad Ibrahim

**Affiliations:** 1 Seed Technology Research Department, Field Crops Research Institute, Agricultural Research Center (ARC), Giza, 12619 Egypt; 2 The Identification of Microorganisms Unit, Plant Pathology Research Institute, Agricultural Research Center (ARC), Giza, 12619 Egypt; 3 Maize and Sugar Crops Diseases Department, Plant Pathology Research Institute, Agricultural Research Center (ARC), Giza, 12619 Egypt; 4 Agricultural Botany Department, Faculty of Agriculture, Cairo University, Giza, 12613 Egypt

**Keywords:** Late wilt disease, *Cephalosporium maydis*, *Trichoderma* spp., Seed priming, Seed coating, Plant extracts, Extra seed power, Anatomy

## Abstract

**Background:**

Late wilt disease, caused by *Cephalosporium maydis*, is one of the most aggressive fungal diseases threatening maize production in Egypt and Mediterranean region. Biological control and pre-cultivation seed treatments are proposed among the best strategies to control *C. maydis* under greenhouse and field conditions. The objective of this study was to evaluate the effectiveness of *Trichoderma* bio-control agents as well as several pre-cultivation seed treatments (priming and coating) in controlling late wilt disease and improving maize production. Five isolates of *C. maydis* were isolated from infected maize plants collected from different Egyptian governorates. In vitro and in vivo experiments were performed to evaluate the efficacy of different treatments in the control of *C. maydis*.

**Results:**

Pathogenicity test revealed that isolate (5) of *C. maydis*, collected from Qalyubia governorate, was the most virulent against the Baladi maize variety. In vitro, five *Trichoderma* isolates (T1, T2, T4, T6, and T7) were the most antagonistic against *C. maydis*. Seed germination tests showed that “extra seed power”– a novel seed treatment– applied by either coating or priming, along with priming with either garlic or moringa extracts significantly outperformed other treatments in enhancing maize germination and seedling parameters. In greenhouse, the lowest significant disease incidence percentages for Giza 168 maize cultivar were achieved with T2, ESP coating, ESP priming, T4, moringa leaf extract priming at 1.0%, Premis Ultra 2.5% fungicide and garlic extract priming at 1.0%, respectively. The same treatments recorded the lowest significant disease incidence percentages for the same maize cultivar under field conditions. The previous results were supported by anatomical investigation of maize stem under different treatments. Moreover, significant improvements in plant height and yield parameters such as ear weight and length, and grain yield were achieved with the same treatments under infection conditions.

**Conclusion:**

Bio-control treatments using *T. asperellum* (T2) and *T. harzianum* (T4) along with seed treatments using ESP by coating and priming were the most effective in reducing late wilt disease incidence and enhancing growth and yield parameters of maize under greenhouse and field conditions.

## Introduction

Maize (*Zea mays* L.) is an important strategic cereal crop in Egypt with cultivated area of 930 thousand hectares and production of 7.5 million tons [1]. Late wilt (black bundle) disease in maize is caused by *Cephalosporium maydis* Samra, Sabet and Hingorani, a soil-borne fungus, which has threatened maize production in Egypt since the 1960s [2]. According to Sunitha et al. [3], Elshahawy and Abd El-Wahed [4] and Hassan et al. [5], late wilt disease is widely spread in the recent time in many temperate and tropical regions of Africa, Asia and Europe. Late wilt disease in maize is so-called due to the retarded appearance of initial disease symptoms until tasseling [3, 6]. In that concern, *C. maydis* penetrates the root system during early growth stages and spreads upwards steadily in xylem vessels, with rapid progression to the fifth internode during anthesis period [4]. External symptoms of late wilt in maize start to appear around 60 days after sowing, involve; gradual discoloration and inward rolling of leaves, followed by leaf dry-out and wilt [3, 4, 7]. Moreover, yellow to reddish brown streaks start to appear on the stalk, which gradually shrinks and wilts with the progress of *C. maydis* infection [3, 4]. Internally, with disease progress, xylem vessels become fully occluded with fungal hyphae and gum-like secretions [4, 7]. Eventually, vascular bundles turn brownish black, while the pith is macerated with yellowish brown color, later becomes hollow [3, 4]. Maize losses due to late wilt disease could reach up to 70% [3–5].

Despite their effectiveness in fungal control, chemical fungicides have posed great threats to environment and human health, beside the growing resistance of fungi to these products [4, 5, 8]. The previous disadvantages urged the need for alternative control methods that are efficient, cost effective, and environmentally safe. In that context, biological control has been proposed among the best strategies for pathogenic control in plants [9]. For example, *Trichoderma* spp. are among mycoparasitic fungi that have been extensively used in the recent decades for fungal control [9]. Moreover, *Trichoderma* spp. are capable of establishing mutualistic relationships with plant species [8, 10]. However, few *Trichoderma* spp. have been investigated until the recent time [9]. Biological control by *Trichoderma* spp. was repeatedly reported for fungal species such as *Fusarium oxysporum* [11–14], *Fusarium solani* [15], and *Rhizoctonia solani* [16, 17] in several plant species. Fewer reports were available on the control of *C. maydis* in maize using *Trichoderma* spp. [6, 18–20].

Pre-cultivation seed treatments have been widely used in the last decades to improve seed germination, enhance growth and yield quantity and quality, and induce tolerance and/or resistance to various biotic and abiotic stress conditions [21]. Seed coating is among seed treatments used to apply beneficial agents such as nutrients, bio-stimulants or protectants to seeds of various economic crops [21]. In that concern, dry powder coating is one of the simplest coating techniques, which can be applied shortly or immediately before cultivation [21]. Seed priming is another seed treatment technique used to enhance germination properties and induce resistance to external environmental threats [22]. This technique involves seed soaking in water and/or growth enhancers or protective agents allowing seeds to imbibe enough water to initiate physiological processes in the embryo without fully hydrating to the extent allowing germination [23]. The major benefits of seed priming include dormancy breaking, acceleration of germination, and reduction of emergence time [22, 23]. Moreover, priming could induce resistance and/or tolerance to various biotic and abiotic stresses through osmoregulation, activation of antioxidant enzyme system, and regulation of stress related proteins [22, 23]. For example, nutrient or osmo-priming using potassium (K) compounds such as K_2_SO_4_, KNO_3,_ K_2_HPO_4_ has been repeatedly reported to enhance germination and improve growth and yield characters under normal and stress conditions for various crops including maize [24–26]. Similarly, plant extracts such as garlic extract have been frequently used in the recent time for seed priming [27, 28]. Seed priming with garlic extract at mild concentrations could be an important bio-stimulator due to its richness in enzymes, proteins, vitamins, minerals, carbohydrates, free sugars, and flavonoids [29]. Moreover, garlic is rich in organo-sulphur compounds such as allicin, diallyl sulfide (DAS), diallyl disulfide (DADS), diallyl trisulfide (DATS) [27, 29]. Such compounds are important allelochemicals, which, not only contribute to growth promotion, but also have an important antifungal activity [30–32]. In a similar manner, seed priming with moringa leaf extract (MLE) has been reported to enhance germination characters and growth performance under various conditions [33–35]. MLE is rich in zeatin (an important cytokinin), vitamins, minerals, phenolic compounds, and antioxidants such as ascorbate and glutathione [28, 33, 36]. The presence of major phenolic compounds in MLE such as quercetin and kaempferol, alkaloids, flavonoids, saponins and tannins contributes to its effectiveness in fungal disease control [37]. Clove (*Syzygium aromaticum*) floral bud and fruit extracts have been also used against various fungal species with high efficacy [38–40]. Antifungal activity of clove is attributed to its major bioactive compounds; eugenol and caryophllene [38–40]. However, no reports were available on seed priming with clove extract. Moreover, no records were found on the antifungal activity of any of the aforementioned plant extracts against *C. maydis*.

The objective of the current investigation is to evaluate the effectiveness of some *Trichoderma* spp. (used as bio-control agents) and various pre-cultivation seed priming and coating treatments (using different compounds and plant extracts) in the control of *C. maydis* causing late wilt disease in maize and enhancement of maize growth and production under infestation conditions.

## Materials and methods

### *C. maydis* isolation, inoculum preparation and pathogenicity determination

Maize plant samples showing typical late wilt symptoms were collected from naturally infected fields located at Giza (29°59′13″N 31°12′42″E), El-Qalyubia (Toukh: 30°21′13.33″N 31°12′7.39″E and Kaha: 30°17′00″N 31°12′00″E) and Kafr-El Sheikh (Sakha: 31°5′12″N 30°56′56.66″E and Kleen: 31°02′47″N 30°51′16″E) governorates, Egypt. Plant sampling was conducted as a part of official missions carried out by different institutes of Agricultural Research Center (ARC) in Egypt to assess the health and welfare of strategically important cultivated crops. Isolation of *C. maydis* was carried out from the lower stem internodes of infected maize plants according to Samra et al. [2]. Five isolates of *C. maydis* (representatives of the five sampling places) were obtained from infected maize plants and were stored at 4 °C for further studies.

The isolates of *C. maydis* were grown into 250 mL potato dextrose broth medium supplemented with 0.2% yeast extract in 500 mL Erlenmeyer flasks according to Zeller et al. [41]. After sterilization, flasks were incubated at 28 ± 2 °C for 2 weeks. The isolated fungi were identified based on cultural, microscopic, and morphological characteristics according to Shoala et al. [42]. A pathogenicity test, using the obtained isolates of *C. maydis*, was conducted on susceptible maize variety (Baladi) seedlings, which were obtained from Field Crops Research Institute, ARC, Giza, Egypt. Seedlings were planted in pots and soil-inoculated with the fungus as described by Hassan et al. [5]. Disease symptoms began to appear approximately 60 days after sowing. Pots were examined at weekly intervals thereafter and symptomatic plants were removed when identified. Fungal isolates were recovered from internodes of symptomatic plants to demonstrate Koch’s postulates. Among the tested isolates, the most virulent isolate was selected and used throughout the present study. The disease incidence (%) was calculated at the end of the trial, 90 days after planting according to Samra et al. [43], as follows: Late wilt disease (%) = [number of dead plants due to *C. maydis* infection during the growing season/total number of examined maize plants] × 100.

### *Trichoderma* spp. isolation

Soil samples were collected from natural heavily infested fields in four Egyptian governorates (Gharbia, Kafr-El Sheikh, Qalyubia and Giza) and were used to isolate antagonistic species. Ten *Trichoderma* isolates were purified and identified according to their morphological features using light microscope at the Unit of Identification of Microorganisms, Plant Pathology Research Institute, Agricultural Research center (ARC), Giza, Egypt [44, 45]. *Trichoderma* isolates were maintained on PDA medium and were stored at 6^°^C (Table 1).

**Table 1:** Source of *Trichoderma* spp. Isolates

No.	Code of isolates	Isolates	Location
1	T1	*Trichoderma* spp.	Kafr-El Sheikh
2	T2	*Trichoderma* spp.	Giza
3	T3	*Trichoderma* spp.	Kafr-El Sheikh
4	T4	*Trichoderma* spp.	Giza
5	T5	*Trichoderma* spp.	Qalyubia
6	T6	*Trichoderma* spp.	Qalyubia
7	T7	*Trichoderma* spp.	Kafr-El Sheikh
8	T8	*Trichoderma* spp.	Gharbia
9	T9	*Trichoderma* spp.	Gharbia
10	T10	*Trichoderma* spp.	Giza

### In vitro experiments

#### Antagonistic effect of *Trichoderma *spp. on* C. maydis* linear growth

The antagonistic effect of the aforementioned *Trichoderma* spp. (Table 1) was tested against the most virulent isolate of *C. maydis*. The virulent isolate of *C. maydis* and different *Trichoderma* spp. isolates were prepared by growing on PDA medium. Dual culture technique was used to measure the antagonistic effect of *Trichoderma* spp. against *C. maydis* [46]. Discs of *C. maydis* and each of the antagonistic *Trichoderma* isolates were inoculated oppositely in the same petri-dish. Each treatment was represented by three petri-dishes as replicates. Control petri-dishes had either *C. maydis* or *Trichoderma* spp. inoculated onto one side of the petri-dish. Inoculated petri-dishes were incubated at 25 ± 2 °C until control plates’ surfaces were completely covered with mycelial growth. At the end of experiment, plates were examined and the linear growth of *C. maydis* was measured to determine the most effective antagonistic isolate of *Trichoderma*. Growth reduction of *C. maydis* was determined by calculating the percentage of mycelial growth inhibition using the following formula:

$$\mathrm{Growth}\;\mathrm{reduction}\;(\%)=\lbrack(\mathrm C-\mathrm T)/\mathrm C\rbrack\times100$$

Where, C is the average linear growth of *C. maydis* in control plates and T is the average linear growth of *C. maydis* with bio-control agent treatments.

#### Assessment of seed treatments on maize germination

##### Seed treatments preparation and application

Ten seed treatments were prepared for in vitro assessment to choose the best among them for greenhouse and field experiments. These treatments were; 1- priming with: K_2_SO_4_ (0.5%), moringa leaf extract (0.5 and 1.0%), clove floral bud extract (0.3, 0.6 and 1.0%), garlic clove extract (0.5 and 1.0%), in addition to a novel seed treatment; extra seed power (ESP) (0.5%), and 2- ESP seed coating. ESP (commercial name) is a newly introduced combination of active compounds such as polyphenols, antioxidants, and micronutrients, and is currently under registration as a patent in the Academy of Scientific Research and Technology, Egypt [47].

For preparation of plant extracts, moringa leaves, clove floral buds and garlic cloves were air-dried, then ground to a powder form. The crude powders were stored in paper bags at room temperature. Before the experiment, moringa, clove, and garlic powders were soaked, each, in distilled water at the ratio of 1:10 (w/v) at room temperature for 24 h to obtain extract stock, then diluted into 100 mL of distilled water to obtain different concentrations [33, 35, 36]. These concentrations were 0.5 and 1.0% for moringa and garlic [28, 48], and 0.3, 0.6 and 1.0% for clove. The obtained extracts were filtered through four layers of cheesecloth, followed by Whatman No.1 filter papers to eliminate fiber debris. Powders of K_2_SO_4_ (0.5 g) and ESP (0.5 g) were diluted into 100 mL of distilled water to obtain concentrations of 0.5% for each. Maize grains (Giza 168 cultivar) were obtained from Maize Research Department, Field Crops Research Institute, ARC, Giza, Egypt. Disinfection of maize grains (Giza 168 cultivar) was done for 5 min using a 0.1% HgCl_2_ solution before rinsing 5–6 times with distilled water. For priming treatments, disinfected maize grains were soaked in each of the abovementioned treatment solutions for 20 h before rinsing with distilled water and air drying for 24 h, after which, they were planted in the laboratory [48]. For ESP coating, disinfected grains were mixed with ESP powder (3 g/100 grains) directly before planting.

##### Seed germination experiments

Germination experiments were conducted at Seed Technology Research Department, ARC, Giza, Egypt, to choose the best treatments for greenhouse and field investigations. Sterilized petri-dishes were used for these experiments. Four replicates of 100 seeds (25 seeds/replicate) for each treatment were planted in 15-cm diameter petri-dishes moistened with distilled water, incubated in a growth chamber at 25 ± 2 ºC and laid in a completely randomized design (CRD). The germination test was performed over a period of 7 days according to ISTA [49]. When a 2 mm radicle had emerged from the seed coat, seeds were considered germinated. Normal seedlings were counted daily and then the germination percentage was calculated [49], in addition to other germination and seedling growth traits. Seed measurements were calculated as follows:

- Germination (G%) = (Number of germinated seeds/Total number of seeds) x 100.
- Germination rate (GR) = a + (a + b) + (a + b + c)…… (a + b + c + m)/n (a + b + c + m). Where each of a, b, c is number of seedlings in the first, second and third count, m is number of seedlings in final count, n is the number of counts.
- Germination speed index (GSI) = ∑ Gt/Dt where (Gt) is the number of germinated seeds on day t and (Dt) is the time corresponding to Gt in days, it was calculated as described by the Association of Official Seed Analysis (AOSA) [50].
- Mean Germination Time (MGT) = Σ Dn/Σn, where (n) is the number of seeds which germinated for the day, (D) is number of days counted from the beginning of germination, it was calculated based on the formula of Kader [51].
- Seedling, shoot and root lengths (cm) were measured with the help of a scale with an average sampling of 10 seedlings for each replicate.
- Seedling fresh and dry weights were measured by electric balance for 10 normal seedlings to determine their fresh weight (g), and then they were dried in a hot-air oven at 85 °C for 12 h to obtain their dry weight (g).
- Seedling Vigor Index I (SVI) = Germination (%) x Total seedling dry weight (g).
- Seedling Vigor Index II (SVII) = Germination (%) x Total seedling length (cm).

### Assessment of bio-control agents and seed treatments in vivo

#### Greenhouse experiment

A pot experiment was conducted in the greenhouse of Plant Pathology Department, The Identification of Microorganisms Unit, ARC, Giza, Egypt during the summer of 2022. Five *Trichoderma* spp. isolates (chosen based on their best antagonistic effect against *C. maydis in vitro*), seven seed treatments (chosen based on seed germination test results), and fungicide (Premis Ultra 2.5%) were tested against the most virulent isolate of *C. maydis* in maize plants. Maize grains of Giza 168 cultivar were obtained from the Field Crops Research Institute, ARC, Giza, Egypt. Grain disinfection was carried out by soaking grains in 5% sodium hypochlorite solution for 3 min, then rinsing in sterile water. The inoculum of *C. maydis* was prepared according to Bertani [52] and Dohroo [53]. Soil infestation was carried out 10 days before planting by mixing *C. maydis* inoculum with sterilized soil (7% formalin) in every pot at the rate of 2–3 g/kg soil (w/w), followed by irrigation. *Trichoderma* treatments were used as spore suspensions (10^7^spores/mL) [54]. Disinfected maize grains were soaked in suspensions of *Trichoderma* treatments for 1 h immediately before planting in pots containing soil infested with *C. maydis*. For seed treatments application, disinfected maize grains were subjected to each chosen treatment according to the detailed procedures mentioned earlier (seed treatments preparation and application), after which they were planted in *C. maydis*-infested soil. For fungicide treatment, Premis Ultra 2.5% (active ingredient: triticonazole) was applied at the rate of 2.0 µL/mL according to Hassan et al. [5]. Each pot was seeded with eight grains, and the plants were thinned to three plants after germination. Nitrogen fertilizer in the form of urea (46% N) was added at the rate of 500 mg N/kg soil, 30 days after planting, and the plants were irrigated when necessary. The experiment was arranged in a randomized complete block design (RCBD). Six pots were used as replicates for each treatment as well as controls. Percentage of dead plants due to *C. maydis* infection was recorded 80 days after planting and used to calculate disease incidence % according to Samra et al. [2] and El-Shafey et al. [55]. The experimental treatments were assigned as follows:

1. Infected control, grain cultivation in soil infested with *C. maydis*.
2. Non-infected control, grain cultivation in non-infested soil.
3. Grain soaking in spore suspension of *Trichoderma* spp. (T1) + cultivation in *C. maydis*-infested soil.
4. Grain soaking in spore suspension of *Trichoderma* spp. (T2) + cultivation in *C. maydis*-infested soil.
5. Grain soaking in spore suspension of *Trichoderma* spp. (T4) + cultivation in *C. maydis*-infested soil.
6. Grain soaking in spore suspension of *Trichoderma* spp. (T6) + cultivation in *C. maydis*-infested soil.
7. Grain soaking in spore suspension of *Trichoderma* spp. (T7) + cultivation in *C. maydis*-infested soil.
8. Grain priming with K_2_SO_4_ (0.5%) + cultivation in *C. maydis*-infested soil.
9. Grain coating with ESP powder (3 g/100 grains) + cultivation in *C. maydis*-infested soil.
10. Grain priming with ESP (0.5%) + cultivation in *C. maydis*-infested soil.
11. Grain priming with MLE (0.5%) + cultivation in *C. maydis*-infested soil.
12. Grain priming with MLE (1.0%) + cultivation in *C. maydis*-infested soil.
13. Grain priming with GE (0.5%) + cultivation in *C. maydis*-infested soil.
14. Grain priming with GE (1.0%) + cultivation in *C. maydis*-infested soil.
15. Grain soaking in fungicide (2.0 µL/mL) + cultivation in *C. maydis*-infested soil.

#### Field experiment

A field experiment was conducted in disease nurseries at Sakha (Kafr-El Sheikh) and Giza (Giza) Research Stations, Plant Pathology Research Institute, ARC, Egypt, from June to October 2023. These nurseries were artificially infested in the previous years with the four clonal lineages of *C. maydis* that cause late wilt of maize in Egypt, which are commonly used in Egyptian maize breeding programs [56]. Maize grains of Giza 168 cultivar were also used in field experiment. They were also obtained from Field Crops Research Institute, ARC, Giza, Egypt. Most of the treatments that were used in greenhouse experiment were involved also in field experiment. Disinfected maize grains were soaked in spore suspensions (10^7^spores/mL) of *Trichoderma* treatments, while seed treatments were applied as previously described. Control grains were soaked in sterile distilled water only. Randomized complete block arrangement in three-replicate plots was used. Plot area was 8.4 m^2^. Each plot had three rows, with a length of 3 m, width of 60 cm, row spacing of 50 cm, and a space between plots of 50 cm. Each plot had 30 maize plants. Grains were sown in hills at depth 5–8 cm (two grains/hill, later thinned to one plant/hill). Recommended irrigation, fertilization, and weed control were practiced. Disease incidence of late wilt was recorded 110 days after sowing as infection percentage. Maize vegetative and yield parameters (final plant height (cm), ear length (cm), ear weight (g), 100-kernel weight (g), and grain yield (ton/feddan)) were evaluated during harvest period. The control treatments used in field experiment are described below:

1. Infected control, grain cultivation in soil infested with *C. maydis*.
2. Grain soaking in spore suspension of *Trichoderma* spp. (T2) + cultivation in *C. maydis*-infested soil.
3. Grain soaking in spore suspension of *Trichoderma* spp. (T4) + cultivation in *C. maydis*-infested soil.
4. Grain priming with K_2_SO_4_ (0.5%) + cultivation in *C. maydis*-infested soil.
5. Grain coating with ESP powder (3 g/100 grains) + cultivation in *C. maydis*-infested soil.
6. Grain priming with ESP (0.5%) + cultivation in *C. maydis*-infested soil.
7. Grain priming with MLE (0.5%) + cultivation in *C. maydis*-infested soil.
8. Grain priming with MLE (1.0%) + cultivation in *C. maydis*-infested soil.
9. Grain priming with GE (0.5%) + cultivation in *C. maydis*-infested soil.
10. Grain priming with GE (1.0%) + cultivation in *C. maydis*-infested soil.
11. Grain soaking in fungicide (2.0 µL/mL) + cultivation in *C. maydis*-infested soil.

### Identification of *Trichoderma* isolates

The most effective *Trichoderma* isolates in greenhouse and field experiments were identified by sequence in GenBank. DNA was extracted using the procedure described by Dellaporta et al. [57] for genomic DNA isolation. The internal transcribed spacer region (ITS) of rRNA was sequenced and amplified using primers ITS4 and ITS5 [58]. The PCR reaction was carried out in a 25-µL reaction volume with 10 µL of PCR Master Mix (amaR OnePCR, GeneDirex, Inc.), 11 µL of ddH_2_O, 1.5 µL of each primer, and 1 µL of template DNA. Sequencing was performed at a specialized sequencing company (Macrogen, Inc., Seoul, Korea). The PCR amplification conditions were carried out following Haouhach et al. [59]. To assign taxonomy, the BLASTn algorithm was performed using the NCBI GenBank database, comparing the queries to type specimens.

### Stem microscopy

Stem samples were taken from the greenhouse experiment at the age of 75 days for healthy and infected plants as well as plants that exhibited reduced disease incidence and wilt symptoms in response to different treatments. Specimens were cut throughout the second and third basal internodes to check for the presence of the fungal hyphae and investigate the structural changes in response to infection and treatments. Preparation was done as described by Nassar and El-Sahhar [60] involving the following steps: fixation in Formaldehyde Aceto-Alcohol (FAA), dehydration in butanol series, embedding in paraffin wax, slicing transversally (20 µ thick) using a rotary microtome, dying using crystal violet-erythrosine double stain and finally mounting in Canada balsam and drying at 45° C. Cross sections of stem were photomicrographed at Cairo University Research Park (CURP), Giza, Egypt, using Leica light image analysis system DM 750. Some histological measurements were taken to compare between different treatments.

### Statistical analysis

Data were analyzed after checking normality of data using one-way analysis of variance (ANOVA) according to Snedecor and Cochran [61]. Data significance was considered at *p* ≤ 0.05. Following ANOVA, means of data were compared using Duncan’s multiple range test [62]. CoStat system for Windows, version 6.311 (CoHort software, Berkeley, CA, USA) was used for statistical analysis of data.

## Results

### *C. maydis* pathogenicity

Results in Table 2 show that the five isolates of *C. maydis* were capable of causing late wilt 110 days after sowing. Non-inoculated plants (control) did not develop late wilt symptoms. Data showed that the tested *C. maydis* isolates varied significantly (*p* ≤ 0.05) in their virulence toward maize plants. The most virulent isolate was no. (5) (82% disease incidence), while the least virulent was isolate no. (1) (65.4% disease incidence).

**Table 2:** Virulence of *C. maydis* isolates against maize Baladi cultivar under greenhouse conditions

Isolate	Location	Disease incidence (%)
**1**	Kafr-El Sheikh (Kleen)	65.4^d^
**2**	Qalyubia (Toukh)	69.2^c^
**3**	Kafr-El Sheikh (Sakha)	72.6^b^
**4**	Giza (Giza)	67.3^cd^
**5**	Qalyubia (Kaha)	82.0^a^
**Control**		0.0^**e**^

^a, b, c, d, e^Means with different letters are significantly different at 0.05 significance level

### In vitro experiments

#### Antagonistic effect of *Trichoderma* spp. *on C. maydis* linear growth

Data in Table 3 show significant variation (*p* ≤ 0.05) in antagonism among the ten studied *Trichoderma* isolates against the most virulent *C. maydis* isolate (isolate 5). *Trichoderma* isolates significantly decreased the mycelial linear growth of *C. maydis* on PDA plates compared with the control. The highest significant reduction in growth of *C. maydis* was obtained with T2 (86.11%) and T4 (83.33%). Meanwhile, T8 and T10 showed the least significant antagonistic effects (67.77 and 68.88%) against *C. maydis* compared to other isolates.

**Table 3:** Antagonistic effect of different *Trichoderma* spp. Isolates on the linear growth of *C. maydis* on PDA medium 7 days after incubation at 25 ± 2 °C

Isolate code	Governorates	*C. maydis*	
		Linear growth	Reduction %
**T1**	Kafr-El Sheikh	1.63^f^	81.88^b^
**T2**	Giza	1.25^h^	86.11^a^
**T3**	Kafr-El Sheikh	2.5^d^	72.22^d^
**T4**	Giza	1.5^g^	83.33^b^
**T5**	Qalyubia	2.5^d^	72.22^d^
**T6**	Qalyubia	2.1^e^	76.66^c^
**T7**	Kafr-El Sheikh	2.0^e^	77.77^c^
**T8**	Gharbia	2.9^b^	67.77^f^
**T9**	Gharbia	2.7^c^	70.00^e^
**T10**	Giza	2.8^bc^	68.88^ef^
**Control**		9^a^	0.00^g^

^a, b, c, d, e, f, g, h^Means with different letters are significantly different at 0.05 significance level

#### Seed treatments effect on maize seed germination

##### Germination characteristics

**Fig. 1:**
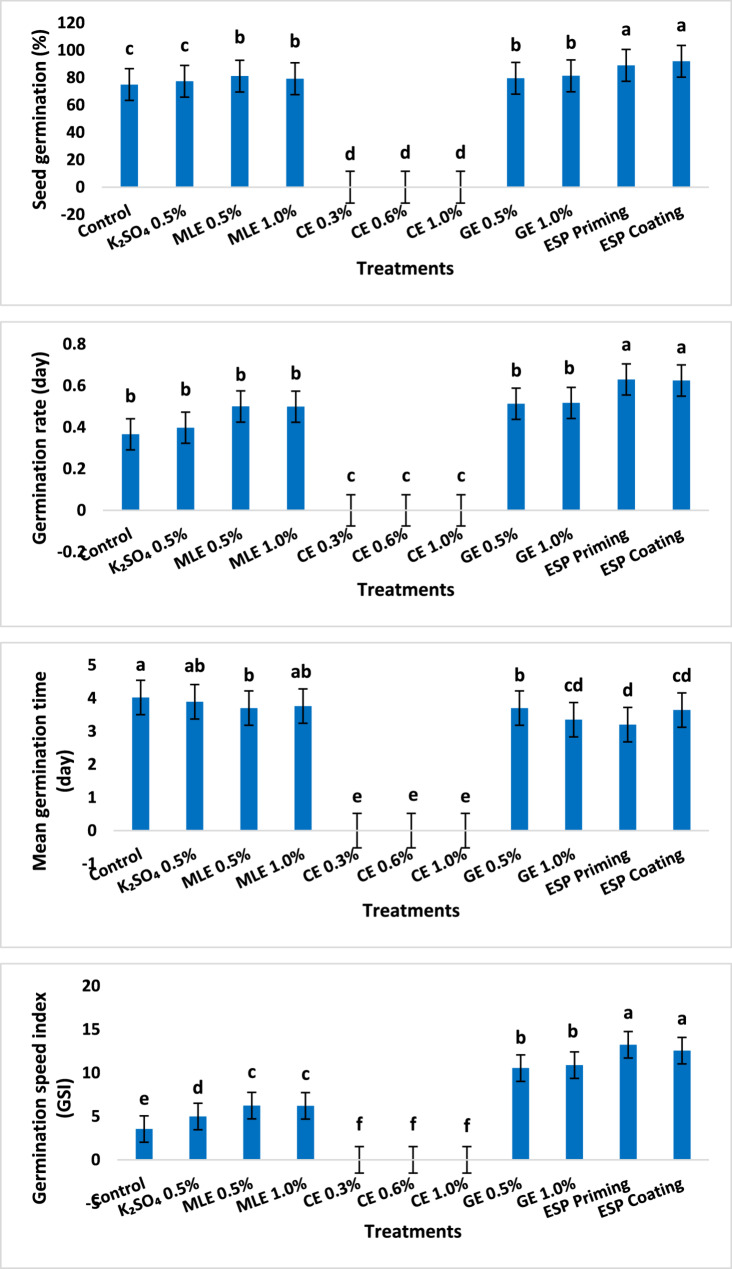
Seed germination parameters of maize (Giza 168 cultivar) under different seed treatments compared to untreated control. Mean with different letters are significantly different at 0.05 significance level

Data demonstrated in Fig. 1 indicate significant differences (*P* ≤ 0.05) among all treatments for all seed germination characters. Worthy to note, no germination or seedling development results were recorded for clove treatments at any of the tested concentrations (0.30, 0.60 and 1.0%) as the seeds failed to germinate. The highest significant values for G % were observed for ESP coating and priming seed treatments (92 and 89%, respectively) followed by moringa (MLE) and garlic (GE) extracts priming treatments at both concentrations (0.5 and 0.1%) while, untreated control recorded the lowest G% for seeds (75%). Compared to control and other treatments, ESP priming and coating recorded the highest significant values of GR/day (0.63 and 0.625, respectively), followed by each of GE and MLE at 1.0 and 0.5% (no significance among these treatments). On the other hand, ESP priming, GE priming at 0.1% and ESP coating treatments recorded the lowest significant MGT (3.2, 3.35, and 3.64 days, respectively) compared to control (4.02 days). Moreover, GSI results indicated that ESP coating and priming treatments recorded the highest significant values (13.23 and 12.56), respectively, followed by GE at 1.0% (10.89) and 0.5% (10.55) compared to control, which recorded the lowest values (3.54). K_2_SO_4_ treatment values were insignificant with those of the control for all the studied germination parameters except for GSI (Fig. 1).

##### Seedling establishment

**Fig. 2:**
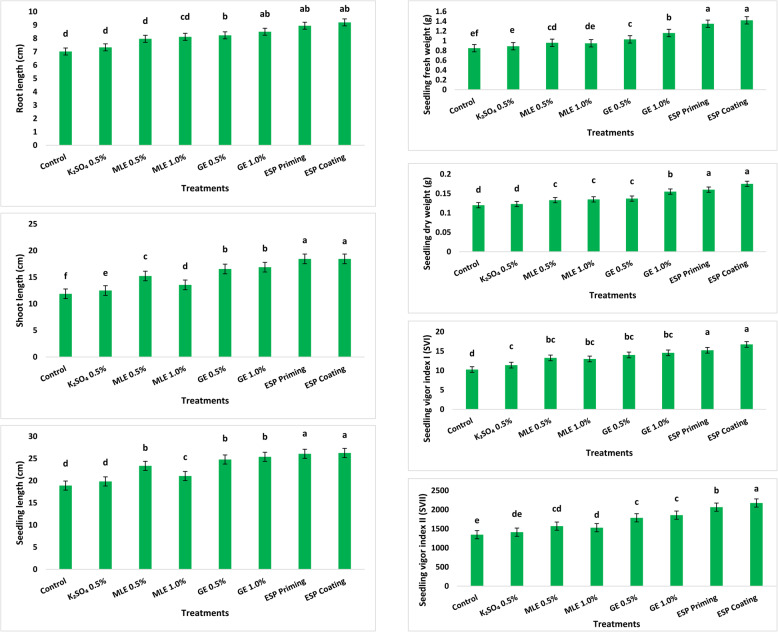
Seedling parameters of maize (Giza 168 cultivar) under different seed treatments compared to untreated control. Mean with different letters are significantly different at 0.05 significance level

Seedling traits were significantly (*P* ≤ 0.05) affected by seed treatments under laboratory conditions (Fig. 2). ESP coating and priming treatments recorded the highest significant increments in seedling (38.9 and 37.9%), root (31.1 and 27.5%), and shoot lengths (55.3 and 55.3%), respectively, compared to untreated control seedlings. GE priming treatments at 0.5 and 1.0% also achieved significant increases in seedling (31.1 and 34.3%), root (17.2 and 21.1%), and shoot lengths (39.3 and 42.2%). The lowest values for seedling, root and shoot lengths were noted for K_2_SO_4_ treatment, which was insignificant with untreated control in terms of root and seedling lengths.

A similar trend was noticed with seedling fresh and dry weights as well as SVI and SVII (Fig. 2). The highest significant increments in seedling fresh (67.1 and 58.8%) and dry (45.8 and 33.3%) weights, SVI (63.3 and 48.6%) and SVII (61.6 and 53.6%) were exhibited by ESP coating and priming treatments, respectively, compared to those of control. GE treatment at 1.0% also exhibited significant increments for seedling fresh (36.5%) and dry (29.2%) weights relative to untreated control. GE treatments at 0.5 and 1.0% were also significantly superior in increasing SVII (32.8 and 38.0%, respectively) over that of control. K_2_SO_4_ treatment was insignificant with control for most of the aforementioned seedling parameters (Fig. 2).

### In vivo experiments

#### Greenhouse experiment

Different control treatments exerted a significant effect (*p* ≤ 0.05) against *C. maydis* under greenhouse conditions (Table 4). All studied treatments significantly decreased late wilt disease incidence compared to infected control. T2, ESP coating and priming, and T4 resulted in the highest significant reduction in disease incidence percentages, respectively. Following these, MLE priming at 1.0%, fungicide, GE priming at 1.0%, and T7 also decreased disease incidence percentage significantly compared to infected control.

**Table 4:** Effect of *Trichoderma* spp. and seed treatments on the incidence of late wilt of maize (Giza 168 cultivar) grown under greenhouse conditions during summer of 2022

Treatments	Disease incidence (%)
**T 1**	28.7^c^
**T 2**	20.5^e^
**T 4**	21.8^e^
**T 6**	30.4^bc^
**T 7**	25.9^d^
**K**_**2**_ **SO**_**4**_ **0.5%**	32.3^b^
**MLE 0.5%**	28.6^c^
**MLE 1.0%**	24.5^d^
**GE 0.5%**	30.1^bc^
**GE 1.0%**	25.6^d^
**ESP priming 0.5%**	21.5^e^
**ESP coating**	20.5^e^
**Fungicide**	25.2^d^
**Infected Control**	82.0^a^
**Non-infected Control**	0.0^f^

^a, b, c, d, e, f^Means with different letters are significantly different at 0.05 significance level

#### Field experiment

Data presented in Table 5 show that all chosen control treatments significantly (*p* ≤ 0.05) reduced late wilt disease incidence under field conditions compared to infected control. Among these, ESP coating, T2, ESP priming and T4 resulted in the highest significant reduction in disease incidence percentages in Giza and Sakha, respectively. Seed priming with MLE at 1.0% and GE at 1.0%, respectively, came in second rank reducing late wilt disease incidence significantly in Giza and Sakha.

**Table 5:** Effect of *Trichoderma* spp. and seed treatments on the incidence of late wilt of maize (Giza 168 cultivar) grown under field conditions at Giza and Sakha research stations, Plant Pathology Research Institute, ARC, Egypt during summer 2023

Treatments	Disease incidence (%)	
	Giza	Sakha
**T 2**	19.9^h^	18.1^g^
**T 4**	21.4^g^	20.6^f^
**K**_**2**_ **SO**_**4**_ **0.5%**	43.1^b^	40.8^b^
**MLE 0.5%**	33.5^d^	31.8^d^
**MLE 1.0%**	25.5^f^	24.6^e^
**GE 0.5%**	36.4^c^	34.0^c^
**GE 1.0%**	26.2^f^	25.3^e^
**ESP priming 0.5%**	20.6^gh^	20.1^f^
**ESP Coating**	19.8^h^	18.2^g^
**Fungicide**	32.2^e^	30.7^d^
**Infected Control**	48.5^a^	46.3^a^

^a, b, c, d, e, f, g, h^Means with different letters are significantly different at 0.05 significance level

### Maize growth and yield parameters

Growth and yield parameters of Giza 168 maize cultivar were significantly affected (*p* ≤ 0.05) by the application of different late wilt control treatments under field conditions (Table 6). Significant increments were achieved by most treatments in growth and yield parameters. ESP coating and priming resulted in the highest significant increments in plant height (32.2 and 29.6%), ear weight (32 and 28.8%) and length (61.9 and 50.4%), 100-grain weight (27.9 and 18.3%), and grain yield (67.3 and 58.9%), respectively, compared to infected control. Highly significant increments were obtained also with T4 treatment over infected control for plant height (29.5%), ear weight (26.4%) and length (49.2%), 100-grain weight (16.1%), and grain yield (55.1%), respectively. Worthy to note that GE treatment at 1.0% recorded high significant increments in plant height (29.6%), ear length (46%), and grain yield (47.7%), while MLE treatment at 1.0% resulted in high significant increments in ear weight (25.4%) and grain yield (48.6%). On the other hand, the lowest increments for growth and yield attributes were found with fungicide treatment, where increments were insignificant with infected control for some yield parameters such ear weight and 100-grain weight.

**Table 6:** Combined effect of different control treatments on vegetative growth and yield attributes of Giza 168 maize cultivar obtained at Giza and Sakha research stations, Plant Pathology Research Institute, ARC, Egypt during summer 2023

Treatments	Plant height (cm)	Ear weight (g)	Ear length (cm)	Hundred grain weight (g)	Grain yield (ton/feddan)
**T 2**	280.6^b^	280.0^cd^	26.56^cd^	37.63^bcd^	3.05^de^
**T 4**	283.52^b^	312.5^b^	30.20^b^	39.49^abc^	3.32^bc^
**K**_**2**_ **SO**_**4**_ **0.5%**	259.0^d^	261.5^e^	24.72^de^	35.90^bcd^	2.88^e^
**MLE 0.5%**	274.4^c^	281.5^cd^	25.30^de^	37.45^bcd^	3.07^de^
**MLE 1.0%**	237.0^f^	310.0^b^	26.79^cd^	38.26^bcd^	3.18^cd^
**GE 0.5%**	246.2^e^	275.0^d^	28.50^bc^	38.00^bcd^	3.08^de^
**GE 1.0%**	283.8^b^	290.0^c^	29.56^b^	38.23^bcd^	3.16^cd^
**ESP priming 0.5%**	283.8^b^	318.5^ab^	30.45^b^	40.23^ab^	3.40^ab^
**ESP Coating**	289.6^a^	326.5^a^	32.76^a^	43.50^a^	3.58^a^
**Fungicide**	238.2^f^	255.3^ef^	23.56^e^	35.70^cd^	2.88^e^
**Infected control**	219.0^g^	247.3^f^	20.24^f^	34.00^d^	2.14^f^

^a, b, c, d, e, f, g^Means with different letters are significantly different at 0.05 significance level

### Identification of *Trichoderma* spp.

The most effective *Trichoderma* isolates in greenhouse and field experiments; T2 and T4, were identified by sequence in GenBank, respectively, as *T. asperellum* with accession number: OQ355565 and *T. harzianum* with accession number: OM757839.

### Maize stem anatomy

**Fig. 3:**
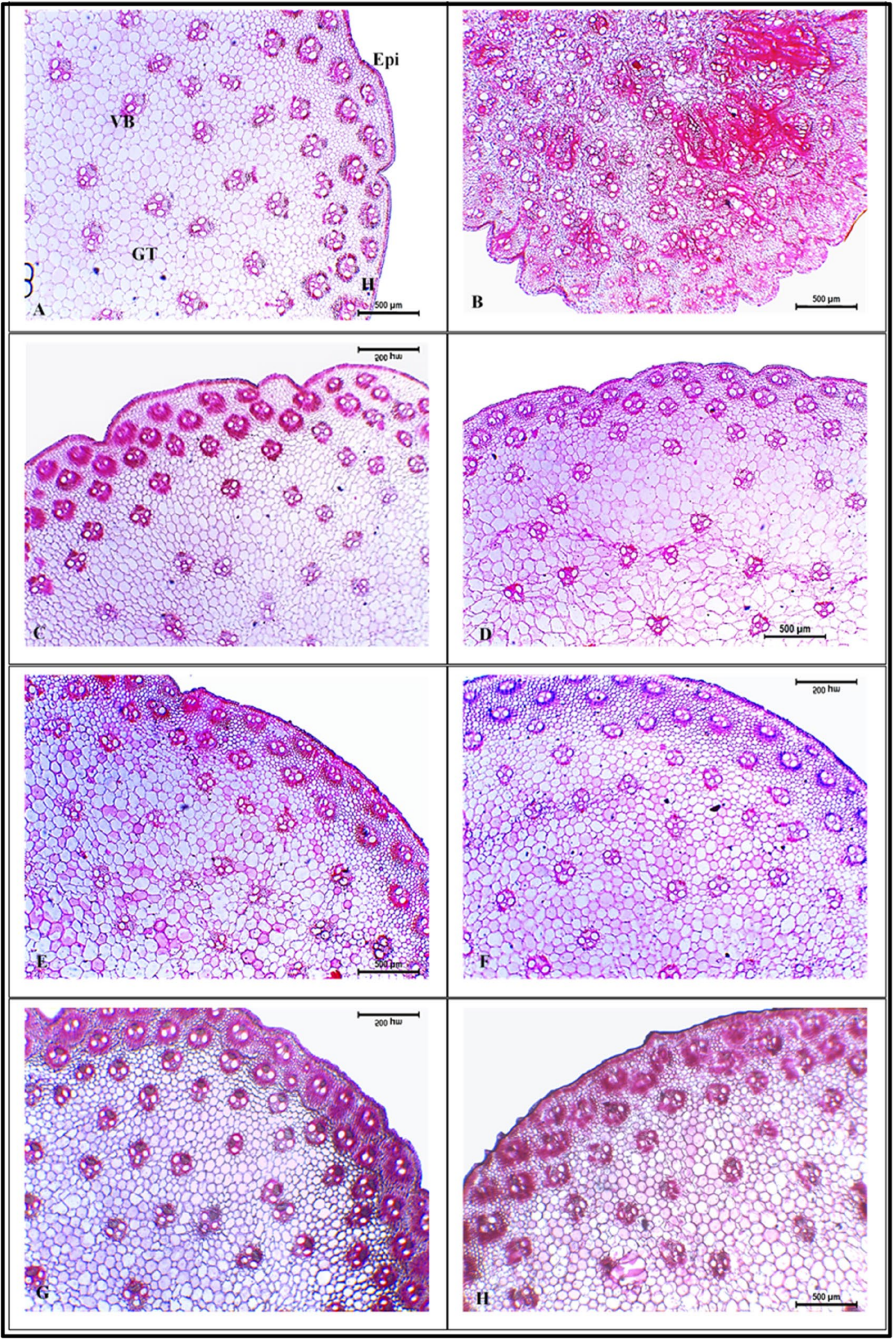
Cross sections of maize stem of plants subjected to different *C. maydis* control treatments under greenhouse conditions. (A) Healthy control, (B) Infected control, (C) Infected with *C. maydis* + T2, (D) Infected with *C. maydis* + T4, (E) Infected with *C. maydis* + MLE at 1.0%, (F) Infected with *C. maydis* + ESP priming, (G) Infected with *C. maydis* + ESP coating, (H) Infected with *C. maydis* + fungicide. Epi: epidermis, H: hypodermis, VB: vascular bundle, GT: ground tissue. Scale bar = 500 μm

**Fig. 4:**
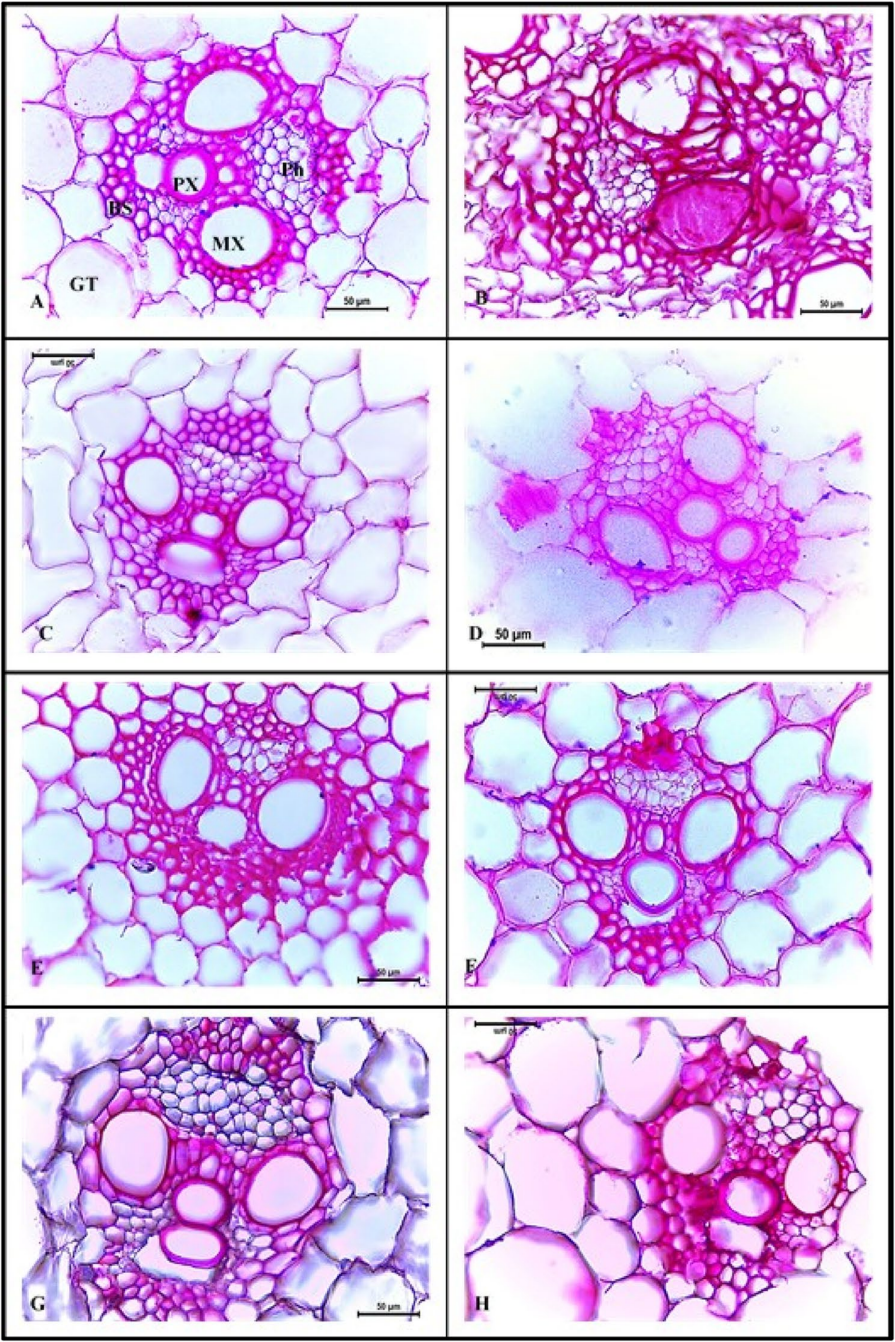
Magnified vascular bundles of maize stem of plants subjected to different *C. maydis* control treatments under greenhouse conditions. (A) Healthy control, (B) Infected control, (C) Infected with *C. maydis* + T2, (D) Infected with *C. maydis* + T4, (E) Infected with *C. maydis* + MLE at 1.0%, (F) Infected with *C. maydis* + ESP priming, (G) Infected with *C. maydis* + ESP coating, (H) Infected with *C. maydis* + fungicide. BS: bundle sheath, MX: meta-xylem, PX: proto-xylem, Ph: phloem, GT: ground tissue. Scale bar = 50 μm

Structural changes of maize stems subjected to infection with *C. maydis* and those with exceeding control treatments are presented in (Figs. 3 and 4). Figures (3B) shows clear deformation in stem of infected plants compared to that of healthy plants (Fig. 3A). In that concern, vascular bundles (VB) of infected stem appeared deformed with disrupted, yet lignified bundle sheath (Figs. 3B and 4B). Moreover, xylem vessels were clogged with gum-like substances and hyphae of *C. maydis* (Fig. 4B). Xylem parenchyma also was hypertrophied (Fig. 4B). Gum-like secretions extend in many areas throughout the stem cross section with completely disrupted ground tissue (Fig. 3B). On the other hand, treatments that showed least disease incidence and exceeding performance in response to infection in greenhouse experiments were T2, T4, ESP coating and priming, MLE priming at 1.0%, and fungicide. For these treatments, transverse sections of stem exhibited a normal anatomical structure, similar to that of control (Figs. 3 and 4: C-H). No signs of *C. maydis* hyphae or gum-like deposits were found in xylem vessels. Structure and distribution of VBs were normal with compact and well-arranged ground tissue. Moreover, significant differences (*p* ≤ 0.05) in histological parameters were noted among the chosen treatments (Table 7). Significant decrements of 26.4 and 20.9% were recorded for stem diameter and phloem thickness of infected stems, respectively, while a significant 13% increment was noted for VB length compared to healthy control plants (Table 7). Furthermore, ESP coating treatment exhibited the highest significant stem diameter (18.4%), VB length (19.3%) and width (3%), and xylem vessel diameter (5.9%), exceeding healthy control plants (Table 7). The ESP priming treatment came in the second rank with a significant increment in stem diameter of 17.2% compared to healthy control plants.

**Table 7:** Histological measurements of maize stem (µm) subjected to different *C. maydis* control treatments under greenhouse conditions

Treatments	Histological parameters (µm)				
	Stem diameter	VB Length	VB Width	Meta-xylem vessel diameter	Phloem thickness
**Control** **(healthy)**	7250^bcd^	215.7^b^	207.9^ab^	59.6^abc^	52.7^a^
**Control** **(infected)**	5333.3^e^	243.7^a^	202.7^b^	63.0^ab^	41.7^b^
**T2**	7583.3^bc^	184.1^c^	173.1^d^	49.5^e^	38.7^b^
**T4**	7000.0^cd^	179.3^c^	196.7^bc^	64.0^a^	39.3^b^
**MLE 0.1%**	7833.3^b^	171.3^c^	204.7^ab^	58.0^bc^	28.2^c^
**ESP priming**	8500.0^a^	212.2^b^	166.8^d^	56.3^cd^	42.4^b^
**ESP coating**	8583.3^a^	257.3^a^	214.2^a^	63.1^ab^	49.8^a^
**Fungicide**	6916.7^d^	223.8^b^	185.5^c^	51.4^de^	42.9^b^

^a, b, c, d, e^Means with different letters are significantly different at 0.05 significance level

## Discussion

*C. maydis* causing late wilt disease is a major threat to maize production in Egypt as well as many countries around Africa, Asia and Europe, with yield losses averaging 40–70% [3–5]. The pathogenicity test performed in the current study revealed that the five *C. maydis* isolates from different Egyptian governorates varied significantly in their virulence, which matches with the results found by Elshahawy and El-Sayed [6] and Hassan et al. [5]. The degree of virulence of *C. maydis* could vary according to geographic distribution and degree of tolerance or susceptibility of maize variety [63]. In that concern, Zeller et al. [41] suggested that *C. maydis* isolates in Egypt could be distinctive to specific geographic regions within Egypt, despite sharing the same genetic background. Moreover, they found that distinctive groups isolated from different Egyptian governorates varied in their virulence. However, further molecular identification of the tested virulent isolates remains essential for better understanding of their phylogeny and genetic variation, thereby offering deeper insights into the management of this serious fungal disease [64].

Chemical fungicides have been used for long time as the most effective method for fungal control [4, 5]. However, their persistence in soil for long periods increase their toxicity to rhizosphere and consequently human health through the consumption of contaminated agricultural products [65]. Moreover, many fungal species have already developed resistance to many fungicidal ingredients [5, 8]. In that perspective, bio-control is considered one good alternative to chemical fungicides [9]. In the current study, the authors investigated the choice of *Trichoderma* spp. for the bio-control of *C. maydis*. The in vitro antagonistic effect showed a significant efficacy of the ten used *Trichoderma* spp., isolated from different Egyptian governorates, against *C. maydis*. However, they varied in their virulence, where the best isolates were T1, T2, T4, T6, and T7. Until the recent time, few *Trichoderma* spp. have been investigated for bio-control [8, 9, 66]. The antagonistic effect of *Trichoderma* spp. against *C. maydis* could be attributed to several mechanisms. The primary mechanism is direct mycoparasitism, in which the antagonistic fungus directly invades and colonizes the hyphae of the pathogenic fungus, breaking its cell wall and resulting in shrinkage of protoplasm and eventually disintegration [9, 66]. Competition over space, water, and nutrients is another mechanism through which *Trichoderma* could affect the pathogenic fungus by weakening and exclusion [8, 66]. Moreover, *Trichoderma* spp. are capable of secreting cell wall hydrolyzing enzymes (chitinases), which causes dissolution of pathogenic fungus cell wall [15, 66]. Antibiosis, through the secretion of antimicrobial secondary metabolites such as trichomycin, trichothecin, trichodermin, and gelatinomycin, is considered also an important mechanism by which *Trichoderma* inhibits the growth of pathogenic fungi [66, 67]. In accordance with the current results, *T. harzianum* and *T. viride* resulted in reduction of linear growth of *C. maydis in vitro* as reported by Elshahawy and El-Sayed [6]. The antagonistic effect of *T. asperellum* on *C. maydis* was reported by Degani et al. [19].

Seed coating and priming are pre-cultivation seed treatments that could break seed dormancy, accelerate germination, enhance seedling establishment and vigor, improve plant growth and quality, and induce resistance to biotic and abiotic stresses [21, 22]. Moreover, their application is very cost-effective [24, 68]. The choice of suitable seed treatments is an important indicator of germination and growth as well as potential resistance to pathogenic attacks. In that concern, the current study investigated the effect of several seed coating and priming treatments on maize grains of Giza 168 cultivar in vitro to assess their efficacy in enhancing germination and seedling traits as well as their potential for controlling *C. maydis* in greenhouse and field experiments. Germination and seedling parameters were found to be significantly enhanced by most treatments. Supreme results were obtained with ESP coating and priming, followed by priming with garlic and moringa extracts. Extra seed power or ESP is the commercial name of a new seed treatment product, which is currently under registration as a patent in the Academy of Scientific Research and Technology, Egypt [47]. ESP powder is a mixture of active ingredients from different plant extracts, such as polyphenols, antioxidants, macro- and micronutrients [47]. In that perspective, priming with macro- and/or micronutrients has been reported to play a significant role in enhancing germination and seedling establishment parameters [68–70]. This is attributed to their vital role in facilitating the uptake and translocation of other nutrients, activating hydrolytic enzymes of food reserves to be available for embryo, synthesis of DNA, RNA, and proteins essential for growth and metabolism, increasing antioxidant enzymes, and decreasing lipid peroxidation [68–70]. In addition, many macro- and micronutrients have a crucial role in the induction of stress resistance [71, 72]. Similarly, priming with organic compounds such as polyphenols could enhance germination characters owing to their ability to promote starch mobilization within the seed, activate the antioxidant enzyme system, scavenge ROS and prevent lipid peroxidation [73, 74]. In addition, priming with antioxidants could accelerate germination by regulating many physiological processes, while also serving as a crucial component of the plant defense system [75]. In consistency with the current findings, improvements of germination and seedling parameters were reported on maize with the application of ESP via seed coating technique [47]. Similarly, GE and MLE are rich sources of macro- and microelements, growth regulators, enzymes, vitamins, antioxidants, phenolic compounds and flavonoids [30, 76]. The previous components are essential for acceleration of seed emergence, enhancement of seedling establishment, growth and yield improvement, and induction of seed resistance to external stresses [27, 34, 35]. In accordance with the current results, improved germination and seedling characters of maize grains by seed priming using GE or MLE were reported by several authors [28, 36, 48, 77, 78].

In greenhouse and field experiments, the two *Trichoderma* isolates T2 and T4 (identified by sequence as *T. asperellum* and *T. harzianum*, respectively) and four seed treatments (ESP coating and priming, and MLE and GE priming at 1.0%) acheived the most significant reduction in late wilt disease incidence percentage in Giza 168 cultivar, surpassing the effect of the tested fungicide. According to Hassan et al. [5], Giza 168 maize cultivar was the most susceptible among other cultivars to infection with *C. maydis*. As mentioned earlier, the capability of *Trichoderma* spp. to reduce pathogenic disease incidence could be attributed to several antagonistic mechanisms [8, 15, 66]. Moreover, *Trichoderma* spp. are capable of inducing systemic resistance in plants through elicitors or cell wall degrading enzymes [66]. In that concern, 10 + elicitors were derived from *Trichoderma* spp. including *T. asperellum* and *T. harzianum* [66]. Additionally, 6-pentyl-α-pyrone, which is a secondary metabolite, was found to be the main active ingredient of *T. asperellum* that is responsible for inhibition of *C. maydis* growth in maize [19]. The findings of the current research matches with those obtained by Elshahawy and El-Sayed [6] and Degani et al. [19] on the bio-control of *C. maydis* in maize using *T. asperellum* and *T. harzianum*, respectively.

The exceeding effect of ESP seed treatment by coating or priming in reducing late wilt disease incidence could be attributed to the combined effect of its ingredients that were described earlier. For example, polyphenols and other antioxidants have been reported to have antifungal activity against several fungal species such as *Candida* and *Fusarium* [79–82]. Several plant polyphenols and antioxidants are capable of inhibition of DNA, RNA, and protein synthesis in fungal cells [80]. Moreover, they have the ability to cause damage to fungal cell wall, interfere with the lipid bilayer of fungal plasma membrane by inhibiting enzymes involved in ergosterol biosynthesis. Lipid peroxidation affects membrane permeability, which results in cellular leakage and eventually apoptosis and cell death [80, 82, 83]. Moreover, they target mitochondrial membrane leading to its destruction and consequently disrupting respiratory functions [79, 81–83]. In addition, polyphenols and other antioxidants are crucial for controlling oxidative stress and activation of plant defense system [80, 83]. The control of *C. maydis* using ESP complex by seed coating or priming is the first to be reported. Seed priming with either GE or MLE at 1.0% was also significantly effective in controlling *C. maydis* and reducing late wilt disease incidence. Antifungal activity of MLE could be attributed mainly to flavonoids such as quercetin and kaempferol [37], in addition to its richness in ascorates, phenolics, alkaloids, saponins and tannins [37]. In a similar manner, organo-sulphur compounds such as allicin, alliin, DAS, DADS, DATS are the major constituents responsible for the antifungal activity of GE [30–32]. Again, this is the first study to report the potential control of *C. maydis* by seed priming treatments using GE and MLE.

Results also revealed that the reduction of late wilt disease incidence was associated with improvement in growth and yield parameters of Giza 168 cultivar under infested field conditions. In that concern, the highest significant improvements in growth and yield parameters were observed the aforementioned bio-control and seed treatments with, ESP coating, ESP priming, and T4 (*T. harzianum*) showing the highest efficacy, followed by T2 (*T. asperellum*), seed priming with GE and MLE at 1.0%. This improvement could be attributed mainly to reduction in disease incidence recorded for the previously mentioned treatments. Moreover, these treatments could have a growth-inducing role, which contributes to the increments of growth and yield parameters. For example, *Trichoderma* spp. could establish mutualistic relationships with plants [10]. In that concern, *Trichoderma* spp. contribute to improvement of rhizoshpere and plant root structure, enhancement of seed germination, increase in photosynthesis, and improved flowering and yield quality [8]. This bio-stimulatory effect and the consequent improvement in productivity and quality of economic crops is attributed to the contribution of *Trichoderma* spp. to synthesis and supply of growth hormone and induction of defense hormone system in plants [8]. In consistency with the current findings, improved growth and yield parameters were recorded by Degani et al. [20] using several *Trichoderma* spp., including *T. asperellum*, for the control of *C. maydis*. Similarly, coating or priming with the nutrient/antioxidant mixture (ESP) and priming with plant extracts (GE and MLE) benefit from the bio-stimulatory effect of their different used components that were discussed earlier. This study is the first to report the role of seed priming or coating with nutrients and/or plant extracts in enhancing growth and yield parameters of maize under *C. maydis* infestation.

The anatomical structure of maize stem is an important indicator of the changes that occurred in response to *C. maydis* infection as well as different control treatments. In that concern, the stem of a healthy maize plant is circular. The outermost layer is epidermis, composed of a single layer of cubic cells. Several layers of sclerenchymatous hypodermis lies beneath the epidermis. Embedded in the hypodermal layer, two rows of closed collateral vascular bundles (CCVB) alternated together in a ring form, after which numerous CCVB are scattered irregularly in the ground tissue. Each VB is surrounded by a sclerenchymatous bundle sheath and is composed of a phloem tissue to the exterior followed by two meta-xylem vessels and one proto-xylem vessel to the interior [84]. On the other hand, infection with *C. maydis* caused an alteration in the stem’s anatomical structure. Deformation and disruption of vascular bundle and ground tissue structures are distinct signs of that infection. Moreover, meta-xylem vessels were clearly plugged by either *C. maydis* hyphae or gum-like secretions. These features are characteristic for *C. maydis* infection and lead to blockage of water transport and eventual wilt symptoms [7]. Similar alterations in maize stem anatomical structure in response to *C. maydis* infection were reported by Ahmed [85] and Ghazy et al. [86]. Lignification of bundle sheath could be regarded as a structural mechanism to delay the progression of fungal infection, as lignin acts as a mechanical barrier against pathogenic invasion [87]. In that concern, Ghazy et al. [86] reported increment in thickness of sclerenchymatous bundle sheath in stems of *C. maydis*-resistant maize cultivars. Similar to the current study, reduction in stem diameter in response to *C. maydis* infection was reported by Ismail [88] while, decrement in phloem thickness was found by Abd El-Rahim et al. [89].

Improvement in stem structure, along with the absence of fungal hyphae and jelly-like occlusions were notable with several treatments. For example, plants treated with Premis Ultra 2.5% fungicide exhibited normal anatomic structure. This could be attributed to the inhibitory effect of its chemical ingrdient, triticonazole, on the mycelial growth of fungus [5, 90] as they interfere with the biosynthesis of ergosterol, which is an essential component of fungal cell membrane [90]. Similarly, treatment with T2 (*T. asperellum*) and T4 (*T. harzianum*) resulted in a normal stem structure due to their antagonistic effect against *C. maydis*. In accordance with the current findings, application of *T. harzianum* in combination with other biocontrol agents enhanced root anatomy of *F. oxysporum*-infected pea plants [13]. Moreover, Ali et al. [15] reported improvement in stem structure of okra plants infected with *Meloidogyne javanica* and *F. solani* with the application of *Trichoderma* spp. In the same manner, seed priming with MLE at 1.0% was also effective against *C. maydis* infection. Normal stem anatomy and absence of fungal hyphae were noticed with that treatment. This could be attributed to the presence of many antimicrobial phytochemicals in MLE, which could act directly against fungus or activate plant defense system via hypersensitivity and lignification [91, 92]. The positive effect of MLE was noticed in the improvement of the anatomical structure of tomato leaves infected with *Alternaria solani* [93]. The best treatment in terms of absence of fungal hyphae as well as enhanced stem anatomical structure and measurements was ESP coating. This treatment resulted in the highest stem diameter and VB dimensions (length and width), while the phloem thickness was equivalent to that of healthy control. Moreover, increased lignification of bundle sheath was also notable with this treatment, indicating more resistant to fungal invasion. The antifungal and bio-stimulatory effect of ESP could be attributed the combined effect of its active components (polyphenols, antioxidants, macro- and micronutrients) described earlier. This study is the first to report the effect of the applied bio-control agents and pre-cultivation seed treatments against *C. maydis* on the anatomical structure of maize stem.

### Future perspectives

The future direction of this study involves addressing several research points that would complement and strengthen the current findings. One key area is the molecular identification of the five *C. maydis* isolates collected from different governorates. This is essential to elucidate the genetic variation among isolates and potentially correlate this variation with their geographic distribution and degree of virulence [41, 63, 64].

Similarly, the *Trichoderma* spp. tested in this study demonstrated strong potential in controlling *C. maydis* under both greenhouse and field conditions. Therefore, advanced molecular identification of these isolates is crucial for accurately assessing and selecting the most effective strains for biocontrol. Furthermore, exploring the specific modes of action by which *Trichoderma* suppresses *C. maydis* could provide valuable insights for future biocontrol strategies.

In addition, seed treatments using plant extracts and the novel product “ESP” were promising in managing late wilt disease and enhancing maize productivity. As such, further investigations into their physiological effects on both the pathogen and the host plant are warranted to better understand their mechanisms and optimize their application in sustainable crop protection strategies.

## Conclusion

Late wilt caused by *C. maydis* is the most virulent disease threatening maize production in Egypt since the 1960s and is currently spreading in many regions of Africa, Asia, and Europe. The current research aimed at evaluating the bio-control of *C. maydis* by *Trichoderma* spp. as antagonistic fungi, as well as the role of several pre-cultivation seed treatments in fungal control. Results of pathogenicity test revealed that *C. maydis* isolate (5), collected from Qalyubia governorate, was the most virulent against the Baladi maize cultivar. In vitro assessment showed that five *Trichoderma* isolates; T1, T2, T4, T6 and T7, were the most antagonistic against the most virulent *C. maydis* isolate. In vitro seed treatments on Giza 168 maize cultivar revealed significant supremacy of ESP coating and priming, as well as GE and MLE priming to other treatments in improving germination and seedling characters. T2 (*T. asperellum*), T4 (*T. harzianum*), ESP coating and priming treatments resulted in the lowest significant late wilt disease incidence percentages in greenhouse and field experiments. GE and MLE priming treatments at 1.0% were also significantly efficient in reducing late wilt disease incidence. Results of stem anatomical investigation supported the effectiveness of these control treatments. Moreover, growth and yield parameters were significantly superior with the same treatments compared to infected plants, highlighting the significant role of *Trichoderma* bio-control agents and pre-cultivation seed treatments as valuable tools for managing late wilt disease in maize. These outcomes encourage the adoption of highly efficient, cost effective and eco-friendly combination of several control strategies as viable alternatives of chemical fungicides in managing fungal diseases.

## Data Availability

Data sharing could be available from the corresponding author upon reasonable request.
